# ﻿Reinstatement of *Cyclocarya serrata* (Juglandaceae) based on ploidy, morphology, niche and phylogenetics

**DOI:** 10.3897/phytokeys.262.155490

**Published:** 2025-09-01

**Authors:** Yan-Feng Song, Quan-Ru Liu, Rui-Min Yu, Da-Yong Zhang, Wei-Ning Bai

**Affiliations:** 1 Ministry of Education Key Laboratory for Biodiversity Science and Ecological Engineering, College of Life Sciences, Beijing Normal University, 100875 Beijing, China Beijing Normal University Beijing China; 2 National Engineering Research Center of Tree Breeding and Ecological Restoration, College of Biological Sciences and Technology, Beijing Forestry University, Beijing 100083, China Beijing Forestry University Beijing China

**Keywords:** *
Cyclocarya
*, morphometric analysis, niches, phylogenetics, polyploidy, taxonomy

## Abstract

*Pterocarya serrata* C.K.Schneid., originally described in 1912, has long been treated as a synonym of *Cyclocarya paliurus* (Batal.) Iljinsk. in Plants of the World Online (POWO). Based on an integrative framework combining ploidy determination, morphometric analyses, ecological niche comparisons, and molecular phylogenetics of mixed-ploidy populations, together with extensive herbarium and field investigations, we formally reinstate *Pterocarya serrata* as a distinct species within the genus *Cyclocarya*, recognizing three synonyms. Our results reveal clear phenotypic divergence and genetic differentiation between *C. serrata* and *C. paliurus*, which have evolved into separate evolutionary lineages. These findings refute the traditional treatment of *Cyclocarya* as a monotypic genus. Furthermore, we present the first comprehensive morphological descriptions, distribution records, and taxonomic notes for both *C. serrata* and *C. paliurus*, thereby advancing the systematic understanding of *Cyclocarya*.

## ﻿Introduction

*Cyclocarya* Iljinsk. comprises a single recognized species, *Cyclocarya paliurus* (Batal.) Iljinsk. (Ilyinskaya 1953; Manning 1975, 1978; Lu et al. 1999; Manos and Stone 2001; POWO 2025). This species is primarily distributed in the moist forests of the East Asian subtropical region, occurring at elevations ranging from 400 to 2500 meters above sea level. Once regarded as a classic example of a monotypic Tertiary relict genus with presumed taxonomic uniformity, *Cyclocarya* has recently been shown through advances in flow cytometry and genomic studies to contain both natural diploid and autotetraploid cytotypes (Wu et al. 2005; Qu et al. 2023; Yu et al. 2023). These findings suggest previously unrecognized genetic and morphological diversity within *Cyclocarya*. Our examination of herbarium specimens and review of historical taxonomic literature suggest that earlier researchers classified individuals within the genus as different species, likely due to morphological differences that, in retrospect, reflect distinctions between diploid and tetraploid cytotypes.

The taxonomic history of *C. paliurus* dates back to Batalin (1893), where the species was originally described under the genus *Pterocarya* as *Pterocarya paliurus* Batalin, based on specimens collected in 1882 near Yichang (Ichang) in Hubei Province and near Ningbo in Zhejiang Province by the Irish plantsman and sinologist Augustine Henry. In the protologue, Batalin described the species as having compound leaves with seven leaflets, each measuring 90–120 mm in length and 40–50 mm in width.

Based on new collections from western Hubei made in 1900 by Wilson from the Arnold Arboretum of Harvard University, Schneider (1912) described *Pterocarya serrata*, explaining that the species differed from *P. paliurus* by its larger leaves, each with 9 to 11 leaflets, which can reach up to 14 cm in length and 3.4 cm in width, with acuminate apices and sharply serrate margins with inward-curving teeth. Additionally, the male inflorescences in Schneider’s new species were elongated, extending up to 16 cm, setting it apart from its close relatives.

Tsoong (1936) introduced *Pterocarya micropaliurus* Tsoong, based on a type specimen from Huangshan, Anhui Province, China. Tsoong described *P. micropaliurus* as differing from its close relative *P. paliurus* Batalin in several key aspects, notably its more numerous but significantly smaller and narrower leaflets (9–11 per leaf), denser and sharper serration, and a shorter raceme (approximately half the length of that in *P. paliurus*). Furthermore, its fruit wings are no more than 2.5 cm in diameter, whereas those of *P. paliurus* are 3–7 cm. In a later taxonomic revision, Hsu et al. (1988) reclassified this species as a variety of *C. paliurus*, renaming it C. paliurus var. micropaliurus (Tsoong) P.S.Hsu, X.Z.Feng & L.G.Xu. Their treatment was based on the observation that *P. micropaliurus* primarily differed from *C. paliurus* in having smaller fruits (wings ~2.5 cm in diameter), more numerous leaflets, and sharper serration.

The current treatment in the *Flora of China* (FOC) (Lu et al. 1999) highlights substantial variation in leaflet number and size, as well as in the shape and dimensions of the fruit wings in *Cyclocarya paliurus*. According to the FOC, certain extreme morphological forms of *C. paliurus* appear sufficiently distinct to warrant recognition as separate species; however, the occurrence of numerous intermediate forms and the lack of clear geographic boundaries were cited in support of treating *C. paliurus* as a single, naturally variable species. Notably, although C. paliurus var. micropaliurus exhibits distinctive morphological traits, it is considered a synonym in the current FOC account. The FOC further lists *Pterocarya paliurus* and *P. micropaliurus* as synonyms under *C. paliurus*, but does not include *Pterocarya serrata* among them. Importantly, the current descriptions do not account for cytotype variations, which suggests a need for further taxonomic clarification.

In this study, we integrated ploidy identification, morphological analyses, genetic assessments and niche comparisons of mixed-ploidy populations to comprehensively evaluate species boundaries and clarify and reinstate the taxonomic status of *C. serrata*. A systematic comparison of morphological traits was conducted across representative specimens of different cytotypes to precisely delineate their taxonomic distinctions, further substantiated by robust molecular evidence. Our findings present a rigorous taxonomic revision, including detailed morphological descriptions and refined distributional records, thereby significantly enhancing the understanding of evolutionary relationships and species delimitation within this group.

## ﻿Materials and methods

### ﻿Sampling and materials collection

**Figure 1.**
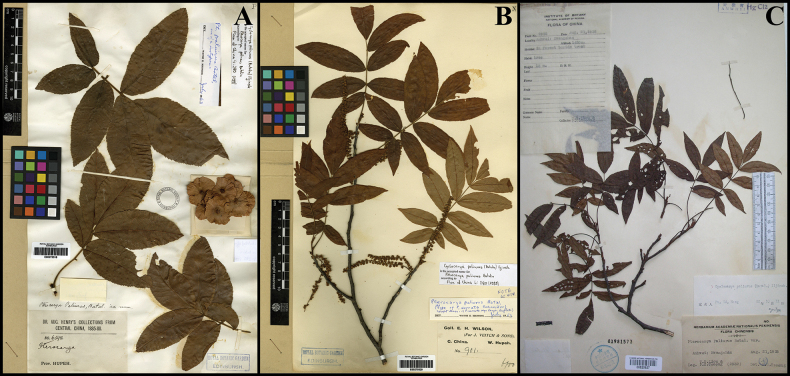
A. Holotype of *Pterocarya paliurus* Batalin (E barcode E00275518); B. Type of *Pterocarya serrata* C.K.Schneid. (E barcode E00275520); C. Holotype of *Pterocarya micropaliurus* Tsoong (PE barcode 00820927).

Between 2020 and 2024, a total of 62 specimens of the genus *Cyclocarya* were collected from various regions across China (see Suppl. materials 1, 2). All specimens provided mature leaf material suitable for detailed morphological measurements and analysis. Additionally, the type specimens of *C. serrata* and *C. paliurus* were sourced from the Royal Botanic Garden Edinburgh (E), the National Herbarium of Victoria (MEL), and The New York Botanical Garden Herbarium (NY), while the type specimen of C. paliurus var. micropaliurus was obtained from the Chinese National Herbarium (PE) (Fig. 1). Morphological measurements included the length and width of the basal, middle, and apical leaflets, the number of leaflets per compound leaf, and the diameter of male flowers.

### ﻿Niche comparisons

We filtered the species distribution data (Suppl. material 3) using the Haversine formula (Prusa and Hill 2021) with a 1 km threshold and converted the cleaned records into a GeoDataFrame (WGS84, EPSG:4326). Additionally, we incorporated diploid samples collected by Qu et al. (2023) from the Huangshan Mountains, further enriching the dataset for analysis. 19 bioclimatic variables and one elevation variable were downloaded from WorldClim 2.1 at a resolution of 30 arc-seconds (Suppl. material 4). Raster values at species occurrence points were extracted using the Python package *Rasterio*, and incomplete records were removed. A Pearson correlation matrix was computed to exclude variables with r > 0.8, and the remaining variables were standardized using Z-score normalization.

We performed principal component analysis (PCA) on the standardized data, retaining the first two components to represent the species’ niche space in two dimensions. Gaussian KDE was then applied to these PCA scores to estimate the density distribution and outline the 50% isopleth. From this, we calculated Schoener’s *D*, which quantifies niche overlap between species, and Levins’ *B*, which assesses niche breadth. Finally, a permutation test with 1000 replicates was performed by randomly shuffling the PCA coordinates to generate a null distribution of Schoener’s *D*, allowing for an assessment of the statistical significance of the observed niche overlap. KDE was applied using a 100 × 100 grid, ensuring a high-resolution representation of species density distributions in the environmental space. All analyses were conducted using Python.

### ﻿Sequencing, ploidy determination and SNP calling

In this study, we newly collected 27 mature leaf samples, which were subsequently resequenced using high-quality next-generation sequencing (NGS) libraries following the protocols described by Yu et al. (2023). Resequencing data for the other 8 diploid and 68 tetraploid individuals were obtained from Yu et al. (2023). Sequencing material from the holotype of *P. micropaliurus* was unavailable, and no samples were collected from the Huangshan region. Although samples from this locality were included in Qu et al. (2023), their resequencing depth was insufficient, and they were therefore excluded from the present analysis. However, we previously obtained a diploid sample from the Dabie Mountains, Jinzhai.

To evaluate ploidy variation, we initially performed flow cytometric analysis on 12 freshly collected individuals out of the 27 newly obtained samples. The detailed methodology is described below.

Ploidy was assessed using a modified OTTO buffer protocol. Approximately 50 mg of fresh leaf tissue was finely chopped in 1 mL of ice-cold OTTO I buffer (100 mM citric acid, 0.5% Tween 20, pH = 3). The homogenate was filtered through a 30 µm nylon mesh to remove debris and centrifuged at 3,000 rpm for 10 min at 4 °C. After discarding the supernatant, the pellet was resuspended in 100 µL of OTTO I buffer, mixed with 500 µL of OTTO II buffer (400 mM Na_2_HPO_4_·12H_2_O, pH = 8), and passed again through a 30 µm nylon mesh. The resulting nuclear suspension was stained with 10 µL of 50 µg/mL propidium iodide (PI) and 10 µL of 50 µg/mL RNase A. After gentle mixing, the mixture was incubated on ice in the dark for 30 minutes prior to analysis. Flow cytometric measurements were performed using a BD Accuri C6 flow cytometer. *Juglans regia* (1C ≈ 606 Mb) was used as the internal reference standard. Due to the limited availability of fresh material, the samples and the standard were processed and analyzed separately (i.e., not co-chopped). Ploidy levels were inferred by comparing the relative fluorescence peak positions of each sample with that of the internal standard.

We further assessed the ploidy levels of the 27 newly sequenced individuals using two complementary computational approaches: nQuire (Weiß et al. 2018), which statistically evaluates allele frequency distributions, and PloidyFrost (Sun et al. 2022), which infers ploidy based on genome-wide topological features derived from De Bruijn graphs.

Sequencing reads were mapped to the published diploid reference genome (https://www.ncbi.nlm.nih.gov/bioproject/PRJNA356989/). In addition, we downloaded raw sequencing data for two diploid reference genomes from Qu et al. (2023) to include them in our phylogenomic analyses. Genome-wide single nucleotide polymorphisms (SNPs) were identified using the variant-calling pipeline described by Yu et al. (2023). To ensure high-quality SNPs, we retained only those with a sequencing depth ranging from at least one-third of the mean sequencing depth to no more than twice the mean depth. To reduce linkage disequilibrium, SNPs were filtered to ensure a minimum inter-site distance of 20 kb across the genome. Variants within coding sequences (CDS), their 2-kb flanking regions, and transposable elements (TE) were excluded to retain putatively neutral sites. Finally, singleton polymorphisms, which are likely due to sequencing errors, were filtered out to minimize false positives.

We obtained two separate joint-called SNP datasets: one dataset contained 3,795 independent SNP loci, while the other comprised 3,084 independent SNP loci and included three outgroup samples (two *Juglans mandshurica* and one *Pterocarya stenoptera*).

### ﻿Mixed-ploidy population genetic analysis

To comprehensively analyze genetic structure of mixed-ploidy populations, we employed multiple analytical approaches. Population genetic structure was assessed using GenoDive v3.06 (Meirmans 2020), which supports mixed-ploidy datasets and integrates both K-means clustering and PCA to reveal genetic patterns among individuals (Azani et al. 2019). Additionally, STRUCTURE v2.3.4 (Pritchard et al. 2000) was used to infer population ancestry under the Admixture model, following the method of Stift et al. (2019), in which individuals in the Di-Tetra mixed-ploidy dataset were coded as tetraploids by inserting two additional lines of missing data (-9). Given the uneven sampling between cytotypes, we adjusted the ALPHA parameter to 0.25. The optimal number of genetic clusters (*K*) was determined using KFinder (Wang 2019), and the results were visualized using the R package *pophelper* (Francis 2017).

### ﻿Phylogenetic reconstruction and genetic differentiation

To reconstruct evolutionary relationships within *Cyclocarya*, we integrated multiple genetic distance and phylogenetic inference approaches. Pairwise genetic distance matrices were generated using the ‘Euclidean’ algorithm implemented in GenoDive v3.06 to quantify genetic differentiation among individuals. These distance matrices were subsequently used to construct a Neighbor-Joining (NJ) tree in the R package *ape* (Paradis and Schliep 2019) for phylogenetic assessment. In addition, pairwise genetic divergence (*D_xy_*) was calculated using *piawka* (Scott et al. 2025), a tool specifically designed to accommodate groups with arbitrary ploidy levels. The resulting *D_xy_* values were visualized as a clustering heatmap using custom Python scripts to investigate patterns of genetic differentiation.

Following Leal et al. (2024), we selected 34 representative individuals spanning both diploid and tetraploid cytotypes for phylogenetic reconstruction. Homologous genomic regions were extracted by generating consensus sequences with bcftools *consensus*, aligning them to the reference genome, and filtering out CDS and transposable element (TE) regions. The retained sequences were divided into 1000 bp fragments at 20-kb intervals; fragments with >50 ambiguous bases (N) were discarded, and monomorphic segments were removed using *SNP-sites* (Keane et al. 2016), yielding 3,490 fragments. Each segment was analyzed independently with IQ-TREE to infer maximum likelihood (ML) phylogenies, which were then integrated using ASTRAL (Zhang et al. 2018) to construct a coalescent-based species tree.

## ﻿Results

### ﻿Ploidy identification

Yu et al. (2023) assembled a chimeric diploid genome and a haplotype-resolved tetraploid genome of *Cyclocarya*, with assembly sizes of 601.46 Mb and 2.36 Gb, respectively.

Ploidy levels of all 12 samples were clearly determined using flow cytometry (Suppl. material 5). These results were subsequently corroborated by the ploidy inferences obtained from nQuire and PloidyFrost. Further analysis using these computational approaches (see Suppl. material 3) revealed that among the 27 newly collected samples, 10 were diploid and 17 were tetraploid, bringing the total to 18 diploid and 85 tetraploid individuals. These results are consistent with our morphological assessments and specimen-based identifications, providing further confirmation that *C. serrata* is diploid, whereas *C. paliurus* is tetraploid.

### ﻿Morphometric analysis

We conducted a morphometric analysis of leaflets and flowers in diploid *C. serrata* and tetraploid *C. paliurus* to assess their morphological divergence. Measurements included 38 apical, 104 lateral, and 37 basal leaflets from 25 *C. serrata* individuals, as well as 47 apical, 92 lateral, and 46 basal leaflets from 23 *C. paliurus* individuals (Suppl. material 6). In addition, 72 male flowers were examined (Suppl. materials 7, 8).

A comparison of leaflet numbers per compound leaf revealed differences between the diploid and tetraploid lineages. Among 38 *C. serrata* compound leaves, 13 exhibited four leaflet pairs, 20 had five pairs, and 5 had six pairs. In contrast, among 47 mature *C. paliurus* compound leaves, 3 had two pairs, 23 had three pairs, and 21 had four pairs, with no instances of five or more leaflet pairs (Suppl. materials 9, 10).

**Figure 2.**
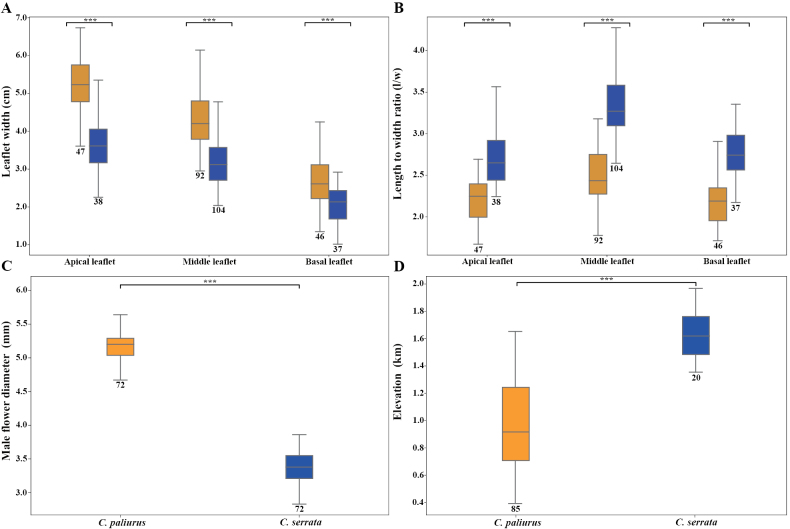
A. Box plot comparing the width of apical, lateral, and basal leaflets between *C. paliurus* and *C. serrata*; B. Comparison of their length-to-width ratios analyzed using the non-parametric Wilcoxon rank sum test; C. Comparison of male flower diameters analyzed using the t-test; D. Altitudinal differences, analyzed using a t-test. Significance is indicated within the figures. (*** = p < 0.001). *C. paliurus* is represented in blue, and *C. serrata* in orange.

Leaflet morphology further distinguished the diploid and tetraploid lineages, with a Wilcoxon rank-sum test applied to untransformed data revealing highly significant differences (p < 0.001) in leaflet width and length-to-width ratios across apical, lateral, and basal leaflets (Fig. 2A, B). *C. paliurus* typically bears 5–11 leaflets, characterized by broader blades with lower length-to-width ratios, ranging from 1.7 to 2.7 in apical leaflets (7.1–17.1 × 3.3–7.3 cm), 1.8 to 3.2 in lateral leaflets (5.7–16.2 × 2.9–6.1 cm), and 1.7 to 2.9 in basal leaflets (2.6–10.1 × 1.3–4.2 cm). The apex is obtuse or acute, and the margin is serrate. In contrast, *C. serrata* generally possesses 9–13 leaflets, with significantly narrower and more elongated blades, exhibiting higher length-to-width ratios of 2.2 to 3.7 in apical leaflets (5.3–12.3 × 2.2–5.3 cm), 2.6 to 4.5 in lateral leaflets (6.5–13.8 × 2–4.7 cm), and 1.8 to 3.8 in basal leaflets (2.7–9.4 × 1–2.9 cm). The apex is acuminate, and the margin is densely denticulate-serrate.

Analysis of male flower diameters revealed further significant morphological differentiation between the species (Fig. 2C). A T-test indicated highly significant differences (p < 0.001), with diameters ranging from 4.46 to 5.76 mm in *C. paliurus* and from 2.83 to 3.86 mm in *C. serrata*, mirroring the trends observed in leaflet dimensions.

Field observations revealed additional ecological distinctions. *C. paliurus* flowers from late April to early May, whereas *C. serrata* flowers from late May through early June. Based on our specimen records, *C. serrata* is distributed at elevations ranging from 1,335 to 1,966 m, whereas *C. paliurus* occurs at elevations between 337 and 1,639 m. Statistical analysis further revealed significant differences in elevation preferences between the two species (p < 0.001), indicating adaptations to distinct ecological niches (Fig. 2D).

These morphometric, statistical, and ecological analyses collectively support the recognition of *C. serrata* as a species distinct from *C. paliurus*.

### ﻿Niche differentiation between *C. paliurus* and *C. serrata*

To reduce multicollinearity, we assessed pairwise correlations among environmental variables and retained nine relatively independent predictors: BIO1, BIO2, BIO3, BIO8, BIO10, BIO12, BIO15, and BIO18. The PCA biplot (Suppl. material 11) displays the separation of *C. paliurus* and *C. serrata* along the first two principal components, which explain 37.2% and 26.7% of the total variance, respectively. Arrows indicate the direction and magnitude of each variable’s contribution. *C. paliurus* exhibits a wider spread along PCA1, while *C. serrata* clusters more tightly in the negative PCA1 space, suggesting species-specific responses to environmental gradients, particularly temperature and precipitation.

**Figure 3.**
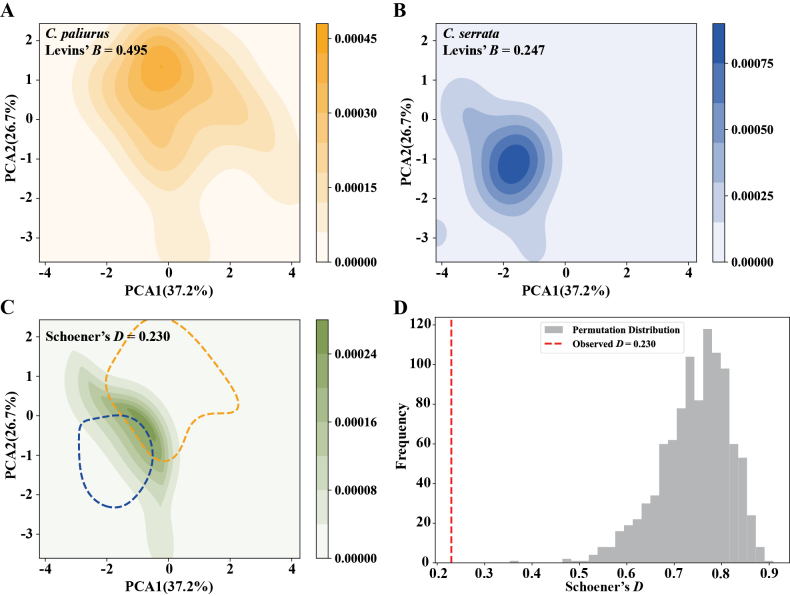
Niche shift patterns of *C. paliurus* and *C. serrata* in environmental space, shown via PCA. A. The ecological niche of *C. paliurus*, with Levins’ *B* indicated; B. The ecological niche of *C. serrata*, with Levins’ *B*; C. Overlap between the two species’ niches (with Schoener’s *D*), dashed contour lines indicate 50% of the background environments, respectively; D. A permutation test on Schoener’s *D*, where the histogram shows the distribution of *D* under random permutations, and the dashed red line marks the observed *D* value.

We further compared the niches of *C. paliurus* and *C. serrata*. The results indicate significant niche expansion and differentiation between the two species. Notably, *C. paliurus* exhibits a broader niche breadth, with a Levins’ *B* value of 0.495, which is approximately twice the value observed in *C. serrata* (0.247) (Fig. 3A, B). Moreover, the degree of niche overlap between the two species is relatively low, with a Schoener’s *D* value of only 0.230 (Fig. 3C). A permutation test further confirms that the observed Schoener’s *D* is significantly lower than expected under a random distribution (Fig. 3D), suggesting that *C. paliurus* and *C. serrata* occupy distinct regions within the environmental space.

### ﻿Molecular phylogenetic analysis

**Figure 4.**
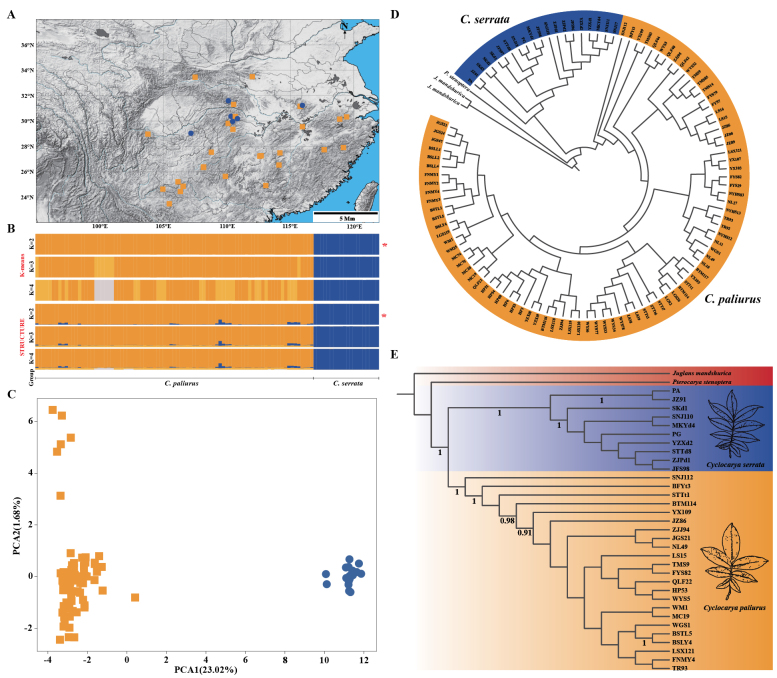
A. The sampling sites of *C. serrata* and *C. paliurus*. Blue dots represent *C. serrata*, while orange squares represent *C. paliurus*; B. Genetic clustering results from STRUCTURE and K-means clustering analyses based on 3,795 independent SNPs, * denotes the best group; C. Principal component analysis (PCA) of the SNP dataset. *C. serrata* shown as blue dots, *C. paliurus* as orange squares; D. Neighbor-Joining (NJ) tree constructed from 3,084 genome-wide independent SNPs, with *C. serrata*, *C. paliurus*, and outgroup species; E. Species tree inferred using ASTRAL from 3,490 genomic intergenic fragment, with *C. serrata*, *C. paliurus*, and outgroup species.

STRUCTURE analysis based on 3,795 independent SNPs identified the best *K* value as 2, clearly separating *C. serrata* and *C. paliurus* into two distinct genetic clusters (Fig. 4A, B, Suppl. material 12). At *K* = 3 or 4, the overall structure remained stable: *C. serrata* consistently formed a single, well-defined cluster, while *C. paliurus* displayed subtle substructure or weak admixture signals in a few individuals. K-means clustering yielded consistent results, also supporting *K* = 2 as the optimal grouping.

PCA (axis 1 explaining 23.02% of the variance) corroborated this species-level differentiation (Fig. 4C). The NJ tree based on 3,084 independent SNPs, the *D_xy_*-based clustering analysis (Suppl. material 13), and the species tree inferred by ASTRAL using 3,490 consensus sequence fragments (35 individuals) all consistently supported the monophyly of *C. serrata* and *C. paliurus*, with all sampled individuals of each species forming a well-supported and clearly separated clade (Fig. 4D, E).

## ﻿Discussion

Owing to the historical neglect of ploidy variation, *C. paliurus* was taxonomically circumscribed in an overly broad sense, encompassing both diploid and tetraploid lineages. In this study, we analyzed and characterized 103 individuals of *Cyclocarya* from 27 populations, assessing differences in ploidy, phenotype, phenology, and elevational distribution. Furthermore, we integrated evidence from molecular phylogenetics and niche comparisons to support the re-instatement of *C. serrata* as a distinct taxonomic entity. Consequently, *Cyclocarya* should no longer be recognized as a monotypic genus.

### ﻿Morphological distinctions between *C. serrata* and *C. paliurus*

Morphologically, *C. serrata* and *C. paliurus* exhibit distinct differences. The compound leaves of *C. serrata* typically bear at least four pairs of leaflets, with some specimens exhibiting up to seven pairs, reaching a total of 15 leaflets in certain cases. In contrast, *C. paliurus* generally possesses no more than four pairs of leaflets, resulting in a significantly lower leaflet count. Additionally, *C. serrata* is characterized by lanceolate leaflets that are markedly narrower than the ovate leaflets of *C. paliurus*, with acuminate apices and densely serrated margins, whereas *C. paliurus* features rounded leaf apices and sparsely serrated margins, further distinguishing the two taxa. Floral morphology also differs substantially, particularly in stamen number, with each male flower of *C. serrata* containing 18–20 stamens, while those of *C. paliurus* bear 30–35 stamens, leading to a larger male flower diameter in the latter. Moreover, Liu et al. (2024) reported significant differences between diploids and tetraploids in multiple morphological traits, including leaflet number, compound leaf area, leaflet length-to-width ratio, leaf shape index, and serration density, reinforcing the morphological divergence between these cytotypes. Such phenotypic differentiation aligns with the well-documented consequences of polyploidization in angiosperms, where genome duplication commonly results in increased cell and organ size (Ramsey and Schemske 2002, Chansler et al. 2016, Jiang et al. 2024).

Environmental pressures may also contribute to morphological variation. Liu et al. (2020) examined leaf traits of three plant species along an elevational gradient and found that both leaf length and width decreased consistently with increasing altitude. Similarly, Dong et al. (2024) reported that, among prickly ash germplasms at comparable latitudes, leaves were smaller in high-altitude regions and larger at lower elevations. In compound-leaved species, leaflets also tend to broaden under low-light conditions to compensate for a reduced leaflet count (Xu et al. 2009). These patterns align with our observations of *C. serrata* and *C. paliurus*: the former, inhabiting higher elevations with intense direct sunlight, exhibits smaller and more numerous leaflets, whereas the latter, typically found in humid, densely forested environments, bears larger but fewer leaflets. Such variation suggests that the phenotypic divergence between the two species may result from the combined influence of genetic and environmental factors.

### ﻿Spatiotemporal niche differences between *C. serrata* and *C. paliurus*

Through niche comparisons, our results demonstrate that polyploids generally conform to the ecological prediction of occupying broader or more diverse niches relative to diploids. This finding aligns with observations from studies on other species complexes, such as *Paspalum intermedium* (Karunarathne et al. 2018), *Dianthus broteri* (López-Jurado et al. 2019), and *Allium oleraceum* (Duchoslav et al. 2020), all of which documented niche expansion or ecological novelty associated with polyploidy.

Specifically, our results reveal that niche differentiation between *C. paliurus* and *C. serrata* primarily involves environmental factors related to temperature and precipitation. Compared to *C. serrata*, *C. paliurus* tends to occupy habitats characterized by warmer temperatures, higher moisture availability, and more pronounced precipitation seasonality. However, the directionality underlying this niche differentiation remains unclear; it is uncertain whether tetraploids expanded progressively into broader ecological niches following whole genome duplication (WGD), or whether diploids experienced niche contraction due to competitive exclusion by tetraploids. Future studies employing fine-scale genomic analyses are needed to elucidate the origin of the tetraploid *C. paliurus* lineage and to clarify the distinct ecological adaptation mechanisms differentiating *C. serrata* and *C. paliurus*.

The *Flora of China* describes the flowering period of *C. paliurus* as spanning from April – May, while the *Flora of Hubei* (Fu 2001) extends it to April – June, and the *Flora of Hunan* (Liu and Wan 2000) records it as May – June. These generalized descriptions likely encompass the combined phenology of both species rather than distinguishing their distinct flowering period. However, our preliminary phenological observations in sympatric regions indicate a clear difference in flowering time between the two species. *C. paliurus* flowers from mid-April to early May, whereas *C. serrata*, which occurs at higher elevations, exhibits a delayed flowering period from mid-May to early June, likely influenced by its montane habitat. This asynchronous flowering pattern suggests a natural mechanism that restricts gene flow between the two species. Such flowering time isolation is widely recognized as a strong prezygotic barrier in plants, playing a crucial role in maintaining species boundaries (Lowry et al. 2008; Matsumoto et al. 2013; Taylor and Friesen 2017; Rose et al. 2024).

### ﻿Genetic divergence between *C. serrata* and *C. paliurus*

Further molecular evidence strongly supports their divergence between *C. paliurus* and *C. serrata*. STRUCTURE analyses reveal two well-defined genetic clusters with no substantial signs of admixture, aside from a weak signal of unidirectional gene flow from the diploid to the tetraploid lineage. Such interploidal unidirectional gene flow from (non-parental) diploids into tetraploids has been documented across a range of systems, including *Triticum aestivum* (Cheng et al. 2019), two *Epidendrum* species (Leal et al. 2025), *Salix* diploid-tetraploid complex (He et al. 2023), and perennial grasses of the genus *Miscanthus* (Clark et al. 2015). Bartolić et al. (2024) suggest that higher ploidy levels tend to be more receptive to genetic material from other cytotypes, which may help mitigate the effects of demographic bottlenecks and inbreeding during the establishment and early expansion of neo-polyploids. Such introgression could introduce critical genetic variation, thereby facilitating population establishment and range expansion, ultimately enhancing the evolutionary potential and long-term persistence of nascent polyploid lineages – exemplified by the broad distribution of *C. paliurus* in contrast to the relictual and isolated refugial populations of *C. serrata*. Consistently, clustering analyses identify two distinct genetic groups, and phylogenetic reconstructions confirm that each forms a well-supported monophyletic lineage. These results further support the interpretation of *C. serrata* and *C. paliurus* as independently evolving taxa with limited genetic exchange. Moreover, our field surveys did not detect any triploid individuals, reinforcing the inference of restricted gene flow and supporting the hypothesis of potential reproductive isolation between the two taxa.

Building on this, evidence from our molecular data suggests that *C. paliurus* most likely originated from a diploid lineage distinct from *C. serrata*. It is possible that this putative diploid ancestor exists but has yet to be sampled in the wild, or it may have gone extinct. Wang et al. (2025) deconstructed the subgenomes of the mixed auto-/allooctoploid *Reynoutria japonica* and identified the presence of unknown or possibly extinct diploid progenitors, highlighting that many polyploid species have ancestral lineages that remain unidentified or have disappeared. Similarly, Ficetola and Stöck (2015) investigated the transgressive niche evolution of the allotetraploid *Bufo pewzowi* and *B. oblongus*, providing further evidence for the extinction of their diploid progenitor. Notably, a recent study in rice by Han et al. (2025) reported that allopolyploids derived from closely related diploid parents can undergo extensive homoeologous exchanges within just a few generations of sexual reproduction. This process may drive what has been termed an “allopolyploid-to-autopolyploid transition.” Therefore, whether *C. serrata* contributed to the origin of *C. paliurus*, or whether other “ghost lineages” were involved, remains an open question that warrants further investigation.

## ﻿Conclusion

Together, our findings suggest that *C. serrata* and *C. paliurus* have evolved as independently diverging lineages, underscoring the value of our integrative approach in uncovering the hidden evolutionary history of polyploid taxa.

## ﻿Taxonomic treatment

### ﻿ Cyclocarya paliurus

Taxon classificationPlantaeFagalesJuglandaceae

(Batal.) Iljinsk., Trudy Bot. Inst. Akad. Nauk S.S.S.R., Ser. 1, Fl. Sist. Vyssh. Rast. 10: 115. 1953.

DA20B52E-3A7D-54FA-B8CC-C7295C35EC58

Fig. 5A–C

≡ Pterocarya paliurus Batalin, Trudy Imp. S.-Peterburgsk. Bot. Sada xiii: 101. 1893.

#### Type.

China • Hubei, *D.A. Henry 6598* (holotype E (E00275518 [image!]), designated by W.E. Manning, 15 Jul. 1963) isotype: MEL (MEL2423219 [image!]), MEL (MEL2423220 [image!])

#### Description.

Trees to 30 m tall. Leaves 20–25 cm; petiole 2.5–5 cm, tomentose or sometimes glabrescent; rachis tomentose; leaflets 5–9, rarely 11, elliptic-ovate to broadly lanceolate, apical leaflets 7.1–17.1 × 3.3–7.3 cm, l/w ratio 1.7–2.7; lateral leaflets 5.7–16.2 × 2.9–6.1 cm, l/w ratio 1.8–3.2; basal leaflets 2.6–10.1 × 1.3–4.2 cm, l/w ratio 1.7–2.9; abaxially pubescent along midvein and secondary veins, base oblique, broadly cuneate to subrounded, apex obtuse or acute, rarely acuminate, margin serrate; terminal petiolule 1–15 mm. Male flowers 4.5–6 mm in diameter, with an entire bract; bracteoles 2; sepals 2; stamens 30–35, anthers pubescent. Female flowers subtended by a small, entire bract, adnate to bracteoles and virtually submerged in wing complex; bracteoles 2, united and adnate to ovary; sepals 4, adnate to ovary, free at apex; style short; stigmas commissural, 2-lobed, plumose. Fruiting spike 25–30 cm, axis glabrous or pubescent. Nutlets compressed globose, ca. 7 mm; disc wing leathery, orbicular to ovate, 6 cm. 2n = 4x = 64.

#### Phenology.

Flowering mid-Apr. – early May; fruiting Jul. – Sep.

#### Distribution and habitat.

*C. paliurus* is widely distributed in the subtropical regions of China, predominantly growing in broad-leaved forests below 1200 m. In northwestern Guangxi, it occurs at elevations exceeding 1600 m.

#### Additional specimens examined.

**Anhui province** • Chiu Hwa Shan, 520 m alt., 2 Aug 1934, *C.S. Fan & Y.Y. Li 69* (NAS barcode NAS00282854) • 11 Spe 1957, *L.G. Fu 913* (HHBG barcode HZ001354, NAS barcode NAS00282870) • Qinyang Xian, 600 m alt., 15 Sep 1987, *W.T. Wang s.n.* (PE barcode 00820925) • Hwang Shan, 700 m alt., 30 Sep 1933, *M. Chen 1200* (CQNM 0001324, PE barcode 00820932) • 600 m alt., 17 Oct 1933, *W.C. Cheng* 4052 (PE barcode 00820926) • 22 Aug 1935, *T.N. Liou & P.C. Tsoong 2946* (PE barcode 00820928) • 25 Oct 1951, *East China Workstation 6163* (NAS barcode NAS00282844, PE barcode 00820934) • 800 m alt., 2 Oct 1955, *M.J. Wang 3601* (NAS barcode NAS00282863, PE barcode 00820922) • Wenquan, 650 m alt., 2 Oct 1955, *M.J. Wang 3676* (NAS barcode NAS00282864, PE barcode 00820923) • 200 m alt., 1 Sep 1957, *L.G. Fu 841* (HHBG barcode HZ001353, NAS barcode NAS00282866) • She Xian, Sanyang, 800 m alt., 13 May 1959, *R.Y. Shan et al. 1623* (NAS barcode NAS00282872) • Yingshan, 13 Jul 1975, *K.J. Guan 75197* (PE barcode 00820931) • Jinzhai, Baimazhai, 950 m alt., 25 May 1986, *K. Yao 9757* (NAS barcode NAS00623146) • Tiantang Zhai, 851 m alt., 26 May 2022, *X.X. Zhu et al. ZXX22471* (KUN barcode 1569870); **Chongqing municipality** • 1000 m alt., 27 Jul 1958, H.F. Zhou 26735 (NAS barcode NAS00282838, PE barcode 00821073) • Fengjie, 800 m alt., 10 Aug 1958, M.Y. Fang 23909 (NAS barcode 0442525, KUN barcode 0442525, PE barcode 00821066) • Nanchuan Xian, 950 m alt., 5 Jul 1957, J.H. Xiong & Z.L. Zhou 91819 (KUN barcode 0442523, PE barcode 00821070); **Fujian province** • Chongan Xian, 300 m alt., 23 Oct 1980, *Wuyi Expedition 802011* (FJIDC barcode FJIDC003052) • Emei Feng, Taining, 1400 m alt., 16 Jun 1945, *L.K. Liu 725* (PE barcode 00821104) • Chuan Ao, Jianning, 600 m alt., 3 Aug 1979, *J.R. Chen et al. 60* (PE barcode 00821105) • Guangze Xian, 750 m alt., 24 Sep 1980, *Wuyi Mountain Expedition 801184* (AU barcode 003911) • Wuyi Shan, Fujian, 1 Sep 1984, *H.Y. Zou 20249* (BJFC barcode BJFC00030313, CSFI barcode CSFI030783); **Guangdong province** • Da Ling Jiao, Ruyuan, 8 Aug 1933, *X.P. Gao 53145* (KUN barcode 0442495, NAS barcode NAS00282833, PE barcode 00821111) • Yam Na Shan, Mei District, 11 Oct 1966, *W.T. Tsang 21366* (NAS barcode NAS00282829, PE barcode 00821108); **Guangxi province** • Tein Hung Shan, 3200 ft [975 m alt.], 11 Aug 1928, *R.C. Ching 6812* (PE barcode 00821114); Lao Shan, Linyun Xian, 13 Jul 1937, *X.Q. Liu 28610* (IBK barcode IBK00155746, NAS barcode NAS00282836, PE barcode 00821113) • Da Wan Shan, Rongshui Xian, 1100 m alt., 10 May 1989, *Beijing Expedition 892540* (PE barcode 00821113) • Yuan Bao Shan, 1200 m alt., 12 Jul 1959, *Q.H. Chang 3646* (PE barcode 00821115) • Leye Xian, 23 May 1977, *D. Fang 3-5817* (GXMI barcode GXMI025397) • Tongle, 1050 m alt., 30 Apr 1989, *Hongshuihe Botanical Expedition 621* (PE barcode 01984063) • Hu Shan, Longsheng Xian, 802.6 m alt., 28 Aug 2014, *Longshengxian Expedition 450328130909002LY* (GXMG barcode GXMG0129711) • Yuanya Shan, Damiaoshan Xian, 1200 m alt., 12 Jul 1959, *Q.H. Lv 3646* (HITBC barcode 030079, IBK barcode IBK00155747, KUN barcode 0442503, NAS barcode NAS00282835); **Guizhou province** • Shunhua, 559 m alt., 16 Jun 2019, *Q.R. Li 52263120190616605LY* (GZTM barcode GZTM0100017) • Xuefeng Shan, Jiangkou Xian, 500–700 m alt., 4 Sep 1986, *Sino-American Guizhou Botanical Expedition 874* (PE barcode 00821078) • Shiqian Xian, 100 m alt., 27 Jul 1988, *WulingShan Expedition 1859* (GFS barcode GFS0016465, KUN barcode 0442489, PE barcode 01352840) • Yinjiang Xian, 1300 m alt., 7 Jul 1988, *WulingShan Expedition 2101* (GFS barcode GFS0005275, KUN barcode 0442492, PE barcode 01352841) • Taiyang Shan, Congjiang Xian, 900 m alt., 22 Aug 1965, *W. Zhang et al. 5-1801* (KUN barcode 0442488, PE barcode 00821094) • 1100 m alt., 14 Jul 1981, *D.F. Huang 01073* (TIE barcode 00002981) • Fanjing Shan, 1600 m alt., 16 May 1959, *T.P. Zhu & Z.F. Liu 787* (KUN barcode 0442485, PE barcode 00821097) • 900 m alt., 8 Apr 1981, *Y.L. Tu 1809* (GNUG barcode GNUG0000248) • Xingren Xian, 1500 m alt., 19 Aug 1960, *Z.S. Zhang & Y.T. Zhang 7897* (NAS barcode NAS00282818, PE barcode 00821085) • Yueliang Shan, Rongjiang Xian, 30 Jul 1959, *Qiannan Expedition 02994* (KUN barcode 0442484, PE barcode 00821096); **Henan province** • Daniu Shan, Shangcheng Xian, 668 m alt., 31 Jul 2021, *X.Z. Cai et al. CXZ531* (HIB barcode HIB0223316); **Hubei province** • Xingshan Xian, 1200 m alt., 30 Aug 2003, *H.D. Huang & H.W. Shi 4539* (HIB barcode HIB0208585) • Li-Chuan, Mo-Pa-Hsiang, 4 May 1948, *C.T. Hwa 492* (PE barcode 00821047) • Hsio-Ho, 3400 ft [1133 m alt.], 4 Sep 1948, *W.C. Cheng & C.T. Hwa 863* (PE barcode 00821046) • Hongqi Xiang, 5 Oct 1957, *G.X. Fu & Z.S. Zhang 1876* (KUN barcode 0442506, NAS barcode NAS00282823, PE barcode 00821050) • 5 Oct 1959, *M.X. Nie & Q.H. Li 1876* (LBG barcode 00053700) • Huangtu Xiang, Laifeng Xian, 560 m alt., 13 Aug 1958, *H.J. Li 4991* (MO barcode 3507492, PE barcode 00821054) • Lvcongpo, Badong Xian, 1200 m alt., 10 Aug 2009, *C.M. Zhao et al. EX3038* (PE barcode 01878087) • Tiantang Zhai, Luotian Xian, 718 m alt., 4 Jul 2022, *X.X. Zhu et al. ZXX22710* (KUN barcode 1566208) • Jiugong Shan, Tongshan Xian, 1146 m alt., 17 Aug 2017, *X.H. Zhan et al. LXP7566* (LBG barcode LBG00136862) • Liushuping Cun, Zhuxi Xian, 1066 m alt., 28 Oct 2017, *S.L. Li GanQL1103* (KUN barcode 1445690); **Hunan province** • Dawei Shan, 1000 m alt., 6 Sep 2016, *H.G. Ye & F.Y. Zeng LXP10-12657* (IBSC barcode 0836509) • 781 m alt., 22 Jun 2017, *K.M. Liu LXP03-07806* (HNNU barcode HNNU00061451) • Yizhang Xian, 800 m alt., 18 Oct 1942, *S.Q. Chen 2614* (IBK barcode IBK00155754, NAS barcode NAS00282824, PE barcode 00821034) • Erping, 685 m alt., 14 Aug 1984, *Q.Z. Lin 84659* (BJFC barcode 00030311, NWFC barcode NWFC0016652) • Xiaoxi Xiang, Yongshun Xian, 20 Jul 2014, *D.G. Zhang zdg1407200834* (JIU barcode JIU04453) • Lijiachong, Xinning Xian, 1100 m alt., 20 Jul 1996, *Z.C. Luo 1506* (PE barcode 00821044) • Fangguang Si, Nan yue, 770 m alt., 12 Oct 1981, *Z.Y. Xie 38* (NYA barcode NYA0004569) • Shadi Ping, Sangzhi Xian, 1150 m alt., 5 Jul 1958, *L.H. Liu 9317* (KUN barcode 0442504, NAS barcode NAS00282827, PE barcode 00821025) • Tongdao Xian, 740 m alt., 19 Oct 1984, *K.W. Liu 33105* (CSFI barcode CSFI030778, NWF barcode CNWFC0016644) • Yun Shan, Wuqing Xian, 1120 m alt., 27 Sep 1963, *L.H. Liu & G.Z. He 16029* (KUN barcode 0442505, PE barcode 00821028) • Goulou Feng, Hengyang, 550 m alt., 23 Aug 1997, *J.B. Zuo 0944* (A barcode 4502650, MO barcode 2669138) • Nan Yue, 22 Aug 1948, *Y. Liu 00499* (PE barcode 00821022, SHM barcode SHM0004435) • Suoxiyu, Cili Xian, 550 m alt., 3 Sep 1984, *Xiangxi Expedition 320* (PE barcode 00821039) • Wukang, Yün-schan, 900 – 1100 m alt., 7 Aug 1917, *H.R.E. Handel-Mazzetti et al*. *11165* (WU barcode 0031431) • Zhupo Cun, Zhijiang Xian, 800 m alt., 18 Oct 1988, *WulingShan Expedition 2325* (PE barcode 00821038) • Yujiaxi, Dayong Xian, 700 m alt., 8 Jul 1985, *Y.T. Xiao 41123* (CSFI barcode CSFI030768) • Tianzi Shan, Zhangjiajie, 1000 m alt., 8 Jul 1985, *H. Zhou & D.S. Zhou 15091003* (CSFI barcode CSFI035146); **Jiangxi province** • Jiuling Shan, Yifeng County, 600 m alt., 8 Jul 1995, *F. Konta & S.J. Hao CH2257* (SHM barcode SHM0016012) • Wuyi Shan, Qianshan Xian, 900 m alt., 15 Apr 2009, *C.M. Tan 09445* (CCAU barcode ccau0006967, JJF barcode JJF00024086, SZG barcode SZG00004480) • Lichuan Xian, 580 m alt., 18 Sep 2015, *H.P. Tong & Y.Z. Wang Tancm2737* (JJF barcode JJF00024100) • Lu Shan, 12 Oct 1951, *M.J. Wang 1147* (KUN barcode 0442507, LBG barcode 00001462, PE barcode 00821012) • Huanglong, 1 Jun 1951, Y. Zou 333 (LBG barcode 00001460, PE barcode 00821011) • Lianhuadong, 27 Sep 1941, *H. Migo* (NAS barcode NAS00282807) • Mumachang, 900 m alt., 1 Jul 1983, *Jin Fang* (BNU barcode 0035041) • Ciping, Suichuan Xian, 900 m alt., 19 May 1977, *J. Xiong 03110* (LBG barcode 00001442, PE barcode 00821010) • Jingang Shan, 1020 m alt., 26 Nov 1963, *J.S. Yue et al. 5422* (NAS barcode NAS00282810, PE barcode 00820996) • Luoxi, Wuning Xian, 1300 m alt., 26 Aug 2000, *J.H. Zhang 1091* (JJF barcode JJF00018768, PE barcode 01569892) • Nanping, Wugong Shan, 1200 m alt., 30 Sep 1954, *Jiangxi Expedition 1647* (NAS barcode NAS00282800, PE barcode 00821006) • Yongxiu Xian, Longgang, 450 m alt., 3 Nov 2010, *Y.Q. Miao 1032* (CCAU barcode ccau0006930, JJF barcode JJF00024092) • Yan Shan, 200 m alt., 9 May 1963, *S.K. Lai 2070* (KUN barcode 0442510, LBG barcode 00108771, PE barcode 00821018, SHM barcode SHM0004442) • Yuanling, Xiushui Xian, 450 m alt., 3 May 2008, *Y.Q. Miao 0822* (JJF barcode JJF00024084, SZG barcode SZG00004474); **Shaanxi province** • Lveyang Xian, 1300 m alt., 16 Sep 1952, *K.J. Fu 5930* (KUN barcode 0442526, PE barcode 00820915); **Yunnan province** • Lida Xiang, Funing Xian, 1150 m alt., 19 Oct 1964, *Q.A. Wu 9667* (KUN barcode 0442480); **Zhejiang province** • Tianmu Shan, 22 May 1929, *K.K. Tsoong 210* (PE barcode 00820971) • 28 Jul 1957, *X.Y. He 25122* (HHBG barcode HZ001332, NAS barcode NAS00282770, PE barcode 00820953) • 25 May 1957, *X.Y. He 21879* (HHBG barcode HZ001340, NAS barcode NAS00282777, PE barcode 00820979) • Changhua, 260 m alt., 15 Jun 1957, *M.B. Deng 4395* (NAS barcode NAS00282785, PE barcode 00820978) • 7 Oct 1957, *X.Y. He 29865* (HHBG barcode HZ001342, NAS barcode NAS00283894, PE barcode 00820951) • W. Tien-mu-shan, 1 Jul 1932, *W.C. Cheng 3687* (PE barcode 00820937) • 350 m alt., 24 Jun 1983, *Q.X. Zheng S815-96* (PE barcode 00820940) • 1040 m alt., 29 Aug 1959, *Zhejiang Botanical Expedition 29287* (NAS barcode NAS00282797, PE barcode 00820955) • Yin xian, 1958, *G.R. Chen 2245* (KUN barcode 0442496, PE barcode 00820989) • Ningp’eh, 17 Jun 1934, *P.C. Tsoong 357* (PE barcode 00820968) • Tiantai, 21 Jul 1959, *L.S. Que 28442* (PE barcode 00820966, ZM barcode ZMNH0000475) • Wuliang Si, Taibai Shan, 260 m alt., 7 Oct 1957, *X.Y. He 29865* (HHBG barcode HZ001342, NAS barcode NAS00282771, PE barcode 00820951) • Lishui, Dagangtou, 29 Jul 1959, *S.Y. Zhang 6109* (AU barcode 031555, HHBG barcode HZ001356, KUN barcode 0442497, NAS barcode NAS00283902, PE barcode 00820945) • Suichang Xian, 30 Oct 1959, *Z.G. Mao 10419* (HHB barcode GHZ001372) • Ruian Xian, 8 Jul 1959, *S.Y. Zhang 6654* (AU barcode 031744, HHBG barcode HZ001355, KUN barcode 0442499, NAS barcode NAS00283914, PE barcode 00820942).

**Figure 5.**
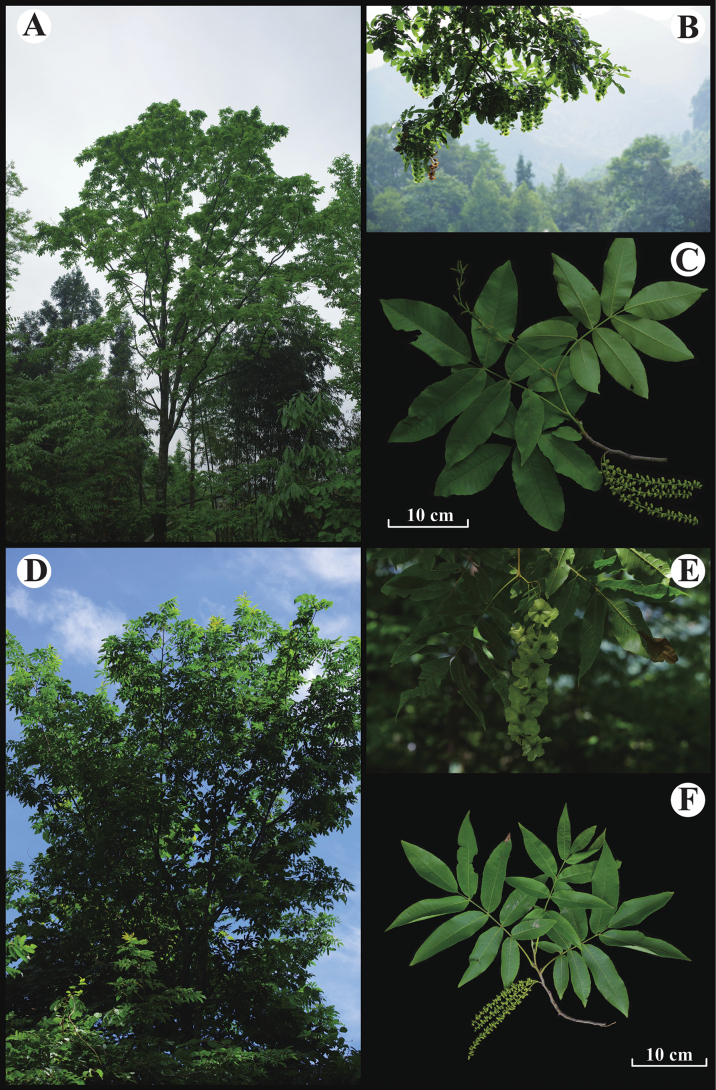
Photographs of tree, fruit, and compound leaf with inflorescence of *Cyclocarya paliurus* (A–C) and *C. serrata* (D–F) (photos by Yan-Feng Song).

### ﻿ Cyclocarya serrata

Taxon classificationPlantaeFagalesJuglandaceae

(C.K.Schneid.) Y.F.Song, W.N.Bai & D.Y.Zhang comb. nov.

EFF647A7-BECD-548F-B830-1BD961B60048

urn:lsid:ipni.org:names:77368453-1

Fig. 5D–F

≡ Pterocarya serrata C.K.Schneid., Ill. Handb. Laubholzk. ii: 880. 1912. Type. E.H. Wilson 901, Western Hubei: Chien-shih-hsien, flowering in June 1900 (lectotype: A barcode A01109592), designated by S.S. Renner & M.S. Dosmann; isolectotypes: A barcode A01109593, E barcode E00275520 (annotated as an isotype of P. serrata by W.E. Manning in July 1963), K barcode K004053893, K barcode K004053899 (annotated as P. paliurus by W.E. Manning in July 1962), LE barcode LE01070443, NY barcode NY00255120 (annotated as an isotype of P. serrata by W.E. Manning in March 1971), NY barcode NY4358432 (annotated as an isotype as P. paliurus by W.E. Manning in March 1971). P barcode P06842578 (annotated as P. paliurus by W.E. Manning in October 1962); Epitype. Fruiting in August 1901, E.H. Wilson 901 (A barcode A01109592), designated by S.S. Renner & M.S. Dosmann; other Wilson 901 collections from August 1900 are preserved at E (barcode E00275519), K (barcode K004053892), P (barcode P06842583), and NY (barcode NY04358771); and K has another undated flowering Wilson 901 sheet (barcode K004053894). Annotation labels for all Wilson 901 collections from both June and August 1900 have been sent to all these herbaria. (Renner and Dosmann 2025)
= Pterocarya micropaliurus Tsoong, Contr. Inst. Bot. Natl. Acad. Peiping 4: 134. 1936. [Pterocarya Micro-Paliurus]. Type. China. Anhui. Huangshan: 1450m alt., 21 Aug. 1935, T.N. Liou & P.C. Tsoong (holotype: PE (PE00820927 [image!]); isotype: IBSC (IBSC0417705); isotype: WUK (WUK0000512)).
≡ Cyclocarya paliurus var. micropaliurus (Tsoong) P.S.Hsu, X.Z.Feng & L.G.Xu, Guihaia 8(4): 322. 1988.

#### Description.

Trees to 30 m tall. Leaves 20–25 cm; petiole 2.5–5 cm, tomentose or sometimes glabrescent; rachis tomentose; leaflets 9–13, long-lanceolate to elongated elliptical, apical leaflets 5.3–12.3 × 2.2–5.3 cm, l/w ratio 2.2–3.7; lateral leaflets 6.5–13.8 × 2–4.7 cm, l/w ratio 2.6–4.5; basal leaflets 2.7–9.4 × 1–2.9 cm, l/w ratio 1.8–3.8; base oblique, apex acuminate, margin densely denticulate-serrate; Male flowers 3–4 mm in diameter, with an entire bract; bracteoles 2; sepals 2; stamens 18–20, anthers pubescent. Female flowers subtended by a small, entire bract, adnate to bracteoles and virtually submerged in wing complex; bracteoles 2, united and adnate to ovary; sepals 4, adnate to ovary, free at apex; style short; stigmas commissural, 2-lobed, plumose. Nutlets compressed globose, ca. 7 mm; disc wing leathery, ovate, 2.5 cm. 2n = 2x = 32.

#### Phenology.

Flowering mid-May – early Jun.; fruiting Aug. – Sep.

Qu et al. (2023) reported diploid individuals from two locations in the Huangshan Mountain Range: Jixi County, Xuancheng (30.127003°N, 118.842934°E, 1406 m alt.) and Huangshan (30.152409°N, 118.167465°E, 1316 m alt.). Their findings, along with the original collection of *Pterocarya micropaliurus* at 1450 m in Huangshan (Tsoong 1936), further support the presence of diploid *C. serrata* at high elevations.

#### Distribution and habitat.

*Cyclocarya serrata* is distributed in the subtropical regions of China, primarily inhabiting broad-leaved forests at elevations above 1300 meters. Its distribution is concentrated from western Hubei (Daba and Wuling Mountains) to eastern Sichuan (Dalou Mountains), with additional occurrences in the Dabie and Huangshan mountain ranges.

#### Additional specimens examined.

**Anhui province** • Huangmaojian Shan, 500 m alt.(?), 22 Jun 2006, *Liu Miao et al. A100046* (KUN barcode 1212700, PE barcode 01800871) • Huang Shan, 23 Sep 1965, *H.X. Zhou 775* (HHBG barcode HZ001348, PE barcode 00820919); **Chongqing municipality** • Nanchuan Xian, Jinfo Shan, 1500 m alt., 6 Jul 2004, *Z.Y. Liu 2040006* (IMC barcode IMC0027823) • 1550 m alt., 14 Jul 1957, *J.H.Xiong & Z.L. Liu 92050* (PE barcode 00821068); **Hubei province** • Shengnongjia, 15 Jul 2017, *D.G. Zhang zdg1816* (JIU barcode JIU36484) • Dajiuhu, 1570 m alt., 2 Aug 2016, *H.D. Huang & D. Wan 525* (HIB barcode HIB0173346) • 1750 m alt., 15 May 2017, *H.D. Huang et al. 1406* (HIB barcode HIB0189791) • Shiliangou, 1500 m alt., 11 Jul 1976, *Shennongjia Expedition 21706* (PE barcode 00990444) • Xiangshuihe, 1940 m alt., 6 Aug 1976, *Shennongjia Botanical Expedition 10986* (PE barcode 00990443) • Xingdou Shan, Lichuan, 1600 m alt., 1981, *G.G. Tang 951* (IBK barcode IBK00155757) • Shennongjia Forest District, 1300 m alt., 14 Sep 1980, *Sino-Amer. Exped. 1299* (MO barcode 3507478, PE barcode 00990437) • Dalaoling, Yichang, 25 May 1977, *Z.A. Wang et al. 216* (CCAU barcode ccau0006964) • 29 May 1977, *X.W. Zhou et al. 003* (CCAU barcode ccau0006969); **Hunan province** • Shimen Xian, 650 – 700 m alt.(?), 9 May 1980, *D.C. Xiao 80275* (CSFI barcode CSFI030772); **Sichuan province** • Leibo, 2174 m alt., 3 Jun 1983, *M.Y. Fang 117877* (PE barcode 02097304).

##### ﻿Determination Key

**Table:** 

1	Leaflets 5–9 per compound leaf, rarely 11; elliptic-ovate to broadly lanceolate; male flowers diameter 4–6 mm; stamens 30–35; Flowering mid-April – early May. (2n = 64)	***C. paliurus***
–	Leaflets 9–13 per compound leaf, rarely 15; long-lanceolate to elongated elliptical; male flowers diameter 3–4 mm; stamens 18–20; Flowering mid-May – early June. (2n = 32)	***C. serrata***

## Supplementary Material

XML Treatment for Cyclocarya paliurus

XML Treatment for Cyclocarya serrata

## ﻿Additional information

### Use of AI

No use of AI was reported.

### Author ORCIDs

Yan-Feng Song https://orcid.org/0000-0003-3121-8443

Quan-Ru Liu https://orcid.org/0000-0003-4270-4746

Rui-Min Yu https://orcid.org/0000-0002-6786-6306

Da-Yong Zhang https://orcid.org/0000-0003-1056-8735

Wei-Ning Bai https://orcid.org/0000-0002-6289-9459

### Data availability

All of the data that support the findings of this study are available in the main text or Supplementary Information.

## Supplementary materials

Supplementary material 1 Specimen collection information, including species, collection number, locality, longitude, latitude, elevation, collector, and collecting date Data type xlsx https://binary.pensoft.net/file/1406755This dataset is made available under the Open Database License (http://opendatacommons.org/licenses/odbl/1.0/). The Open Database License (ODbL) is a license agreement intended to allow users to freely share, modify, and use this Dataset while maintaining this same freedom for others, provided that the original source and author(s) are credited.Yan-Feng Song, Quan-Ru Liu, Rui-Min Yu, Da-Yong Zhang, Wei-Ning Bai

Supplementary material 2 Voucher specimens in this study Data type docx https://binary.pensoft.net/file/1406756This dataset is made available under the Open Database License (http://opendatacommons.org/licenses/odbl/1.0/). The Open Database License (ODbL) is a license agreement intended to allow users to freely share, modify, and use this Dataset while maintaining this same freedom for others, provided that the original source and author(s) are credited.Yan-Feng Song, Quan-Ru Liu, Rui-Min Yu, Da-Yong Zhang, Wei-Ning Bai

Supplementary material 3 Ploidy estimation results from nQuire and Ploidyfrost, geographic coordinates, elevation, and data source for each sample Data type xlsx https://binary.pensoft.net/file/1406757This dataset is made available under the Open Database License (http://opendatacommons.org/licenses/odbl/1.0/). The Open Database License (ODbL) is a license agreement intended to allow users to freely share, modify, and use this Dataset while maintaining this same freedom for others, provided that the original source and author(s) are credited.Yan-Feng Song, Quan-Ru Liu, Rui-Min Yu, Da-Yong Zhang, Wei-Ning Bai

Supplementary material 4 Twenty environmental variables from WorldClim, including 19 bioclimatic variables and one elevation variable Data type xlsx https://binary.pensoft.net/file/1406758This dataset is made available under the Open Database License (http://opendatacommons.org/licenses/odbl/1.0/). The Open Database License (ODbL) is a license agreement intended to allow users to freely share, modify, and use this Dataset while maintaining this same freedom for others, provided that the original source and author(s) are credited.Yan-Feng Song, Quan-Ru Liu, Rui-Min Yu, Da-Yong Zhang, Wei-Ning Bai

Supplementary material 5 Flow cytometric histograms showing the relative PI fluorescence intensity in nuclei from leaves of 12 *Cyclocarya* samples, with *J. regia* as the internal reference Data type pdf https://binary.pensoft.net/file/1406759This dataset is made available under the Open Database License (http://opendatacommons.org/licenses/odbl/1.0/). The Open Database License (ODbL) is a license agreement intended to allow users to freely share, modify, and use this Dataset while maintaining this same freedom for others, provided that the original source and author(s) are credited.Yan-Feng Song, Quan-Ru Liu, Rui-Min Yu, Da-Yong Zhang, Wei-Ning Bai

Supplementary material 6 Length, width, and length-to-width ratio measurements of apical, lateral, and basal leaflets in *C. paliurus* and *C. serrata* (cm) Data type xlsx https://binary.pensoft.net/file/1406760This dataset is made available under the Open Database License (http://opendatacommons.org/licenses/odbl/1.0/). The Open Database License (ODbL) is a license agreement intended to allow users to freely share, modify, and use this Dataset while maintaining this same freedom for others, provided that the original source and author(s) are credited.Yan-Feng Song, Quan-Ru Liu, Rui-Min Yu, Da-Yong Zhang, Wei-Ning Bai

Supplementary material 7 Diameter measurements of male flowers in *C. paliurus* and *C. serrata* (mm) Data type xlsx https://binary.pensoft.net/file/1406761This dataset is made available under the Open Database License (http://opendatacommons.org/licenses/odbl/1.0/). The Open Database License (ODbL) is a license agreement intended to allow users to freely share, modify, and use this Dataset while maintaining this same freedom for others, provided that the original source and author(s) are credited.Yan-Feng Song, Quan-Ru Liu, Rui-Min Yu, Da-Yong Zhang, Wei-Ning Bai

Supplementary material 8 Male flowers of *C. paliurus* and *C. serrata* Data type pdf https://binary.pensoft.net/file/1406762This dataset is made available under the Open Database License (http://opendatacommons.org/licenses/odbl/1.0/). The Open Database License (ODbL) is a license agreement intended to allow users to freely share, modify, and use this Dataset while maintaining this same freedom for others, provided that the original source and author(s) are credited.Yan-Feng Song, Quan-Ru Liu, Rui-Min Yu, Da-Yong Zhang, Wei-Ning Bai

Supplementary material 9 Number of leaflets per compound leaf in *C. paliurus* and *C. serrata* Data type xlsx https://binary.pensoft.net/file/1406763This dataset is made available under the Open Database License (http://opendatacommons.org/licenses/odbl/1.0/). The Open Database License (ODbL) is a license agreement intended to allow users to freely share, modify, and use this Dataset while maintaining this same freedom for others, provided that the original source and author(s) are credited.Yan-Feng Song, Quan-Ru Liu, Rui-Min Yu, Da-Yong Zhang, Wei-Ning Bai

Supplementary material 10 Bar chart of leaflet numbers, with orange representing *C. paliurus* and blue representing *C. serrata* Data type pdf https://binary.pensoft.net/file/1406764This dataset is made available under the Open Database License (http://opendatacommons.org/licenses/odbl/1.0/). The Open Database License (ODbL) is a license agreement intended to allow users to freely share, modify, and use this Dataset while maintaining this same freedom for others, provided that the original source and author(s) are credited.Yan-Feng Song, Quan-Ru Liu, Rui-Min Yu, Da-Yong Zhang, Wei-Ning Bai

Supplementary material 11 PCA plot showing climatic niche differentiation between the two species Data type pdf https://binary.pensoft.net/file/1406765This dataset is made available under the Open Database License (http://opendatacommons.org/licenses/odbl/1.0/). The Open Database License (ODbL) is a license agreement intended to allow users to freely share, modify, and use this Dataset while maintaining this same freedom for others, provided that the original source and author(s) are credited.Yan-Feng Song, Quan-Ru Liu, Rui-Min Yu, Da-Yong Zhang, Wei-Ning Bai

Supplementary material 12 The parsimonious estimate of the number of populations from STRUCTURE analysis using Kfinder, and the population clustering results using K-means Data type txt https://binary.pensoft.net/file/1406766This dataset is made available under the Open Database License (http://opendatacommons.org/licenses/odbl/1.0/). The Open Database License (ODbL) is a license agreement intended to allow users to freely share, modify, and use this Dataset while maintaining this same freedom for others, provided that the original source and author(s) are credited.Yan-Feng Song, Quan-Ru Liu, Rui-Min Yu, Da-Yong Zhang, Wei-Ning Bai

Supplementary material 13 Clustering heatmap based on *D_xy_* values, with orange and blue clades representing *C. paliurus* and *C. serrata*, respectively Data type pdf https://binary.pensoft.net/file/1406767This dataset is made available under the Open Database License (http://opendatacommons.org/licenses/odbl/1.0/). The Open Database License (ODbL) is a license agreement intended to allow users to freely share, modify, and use this Dataset while maintaining this same freedom for others, provided that the original source and author(s) are credited.Yan-Feng Song, Quan-Ru Liu, Rui-Min Yu, Da-Yong Zhang, Wei-Ning Bai
