# Supplementary material for: ﻿Reinstatement of Cyclocarya serrata (Juglandaceae) based on ploidy, morphology, niche and phylogenetics
Source: PhytoKeys. 2025 Sep 1;262:45–71. doi: 10.3897/phytokeys.262.155490 (PMC12418027; doi:10.3897/phytokeys.262.155490)
Supplement: Supplementary material 2 — Voucher specimens in this study [file phytokeys-262-045_article-155490__-s002.docx]

**Suppl. material 2. Voucher specimens in this study.**

| Taxon name, locality, date, *collector*, *collector number* (herbarium barcode) |
| --- |
| ***Cyclocarya paliurus* (Batal.) Iljinsk**., Hubei, Hefeng, 8 Aug 2023, *Y.F. Song*, *BFY3* (BNU barcode 0065645, PEY barcode 0068738); ***C. paliurus***, Guangxi, Longlin, 8 Jul 2024, *Y.F. Song*, *BSLL1* (BNU barcode 0065642, PEY barcode 0068725); ***C. paliurus***, Guangxi, Tianlin, 15 Jul 2024, *Y.F. Song*, *BSTL5* (BNU barcode 0065643, PEY barcode 0068734); ***C. paliurus***, Henan, Nanzhao, 12 Aug 2021, *R.M. Yu*, *BTM114* (BNU barcode 0065636, PEY barcode 0068739); ***C. paliurus***, Zhejiang, Longquan, 4 Aug 2021, *R.M. Yu*, *FYS29*, *FYS79* (PEY barcode 0068735; BNU s.n.); ***C. paliurus***, Guangxi, Lonsheng, 31 Jul 2021, *W.P. Zhang*, *HP51* (BNU barcode 0065654); ***C. paliurus***, Jiangxi, Ji'An, 4 Jul 2020, *W.P. Zhang*, *JGS21*, *JGS47* (BNU barcode 0065655; BNU s.n.); ***C. paliurus***, Anhui, Jinzhai, 20 Jun 2020, *W.P. Zhang*, *JZ86*, *JZ89* (BNU barcode 0065659; BNU s.n.); ***C. paliurus***, Guizhou, Qiandongnan, 12 Sep 2021, *W.P. Zhang*, *LGS* (BNU barcode 0065646); ***C. paliurus***, Jiangxi, Jiujiang, 18 Jul 2020, *W.P. Zhang*, *LS16*, *LS38* (BNU s.n.); ***C. paliurus***, Guangdong, Shaoguan, 20 Jul 2020, *W.P. Zhang*, *NL* (BNU barcode 0065641); ***C. paliurus***, Anhui, Xuancheng, 1 Aug 2021, *R.M. Yu*, *QLF44* (BNU s.n.); ***C. paliurus***, Hubei, Shennongjia, 12 Aug 2022, *R.M. Yu*, *SNJ112* (BNU barcode 0065640); ***C. paliurus***, Hubei, Wufeng, 4 Aug 2023, *Y.F. Song*, *STT1*, *STT3*, *STT6*, *STT7* (BNU s.n.; BNU s.n.; BNU barcode 0065657, PEY barcode 0068736; BNU barcode 0065647, PEY barcode 0068741); ***C. paliurus***, Zhejiang, Lin'An, 2 Aug 2021, *R.M. Yu*, *TMS9*, *TMS14* (BNU s.n.; BNU s.n.); ***C. paliurus***, Guizhou, Tongren, 28 Aug 2021, *W.P. Zhang*, *TR92* (BNU barcode 0065637); ***C. paliurus***, Jiangxi, Pingxiang, 16 Jul 2024, *Y.F. Song*, *WGS1* (BNU s.n.); ***C. paliurus***, Guizhou, Wangmo, 15 Sep 2021, *W.P. Zhang*, *WM25* (BNU s.n.); ***C. paliurus***, Fujian, Nanping, 24 Jun 2020, *W.P. Zhang*, *WYS32*, *WYS33* (BNU barcode 0065653; PEY barcode 0068740); ***C. paliurus***, Shaanxi, Yang, 20 Aug 2021, *R.M. Yu*, *YX103* (BNU barcode 0065635); ***C. paliurus***, Hubei, Hefeng, 5 Aug 2023, *Y.F. Song*, *YZX8*, *YZX9* (BNU barcode 0065658, PEY barcode 0068737; PEY barcode 0068731); ***C. paliurus***, Hunan, Zhangjiajie, 25 Aug 2021, *W.P. Zhang*, *ZJJ94* (BNU barcode 0065639); ***Cyclocarya serrata* (Schneid.) Y.F.Song, W.N.Bai & D.Y.Zhang** , Chongqing, Nanchuan, 3 Sep 2021, *W.P. Zhang*, *JFS96*, *JFS97*, *JFS98* (BNU barcode 0065638; BNU s.n.; BNU s.n.); ***C. serrata***, Anhui, Jinzhai, 20 Jun 2020, *W.P. Zhang*, *JZ91* (BNU s.n.) ***C. serrata***, Hubei, Wufeng, 5 Jun 2024, *Y.F. Song*, *MKY1*, *MKY4* (PEY barcode 0068732; PEY barcode 0068733); ***C. serrata***, Hubei, Wufeng, 22 May 2024, *Y.F. Song*, *SK1*, *SK2*, *SK3* (BNU barcode 0065644, PEY barcode 0068724; BNU barcode 0065651, PEY barcode 0068726; PEY barcode 0068728); ***C. serrata***, Hubei, Wufeng, 4 Aug 2023, *Y.F. Song*, *STT8* (BNU barcode 0065648, PEY barcode 0068730); ***C. serrata***, Hubei, Shennongjia, 14 Aug 2022, *R.M. Yu*, *SNJ17*, *SNJ18*, *SNJ110*, *SNJ111* (BNU s.n.; BNU s.n.; BNU barcode 0065656, PEY barcode 0068727; BNU barcode 0065660); ***C. serrata***, Hubei, Hefeng, 5 Aug 2023, *Y.F. Song*, *YZX1*, *YZX2* (BNU barcode 0065649; BNU barcode 0065650); ***C. serrata***, Hubei, Hefeng, 9 Aug 2023, *Y.F. Song*, *ZJP1*, *ZJP2* (BNU barcode 0065652; PEY barcode 0068729). |
