# Supplementary figures and images for: ﻿Reinstatement of Cyclocarya serrata (Juglandaceae) based on ploidy, morphology, niche and phylogenetics
Source: PhytoKeys. 2025 Sep 1;262:45–71. doi: 10.3897/phytokeys.262.155490 (PMC12418027; doi:10.3897/phytokeys.262.155490)

**A**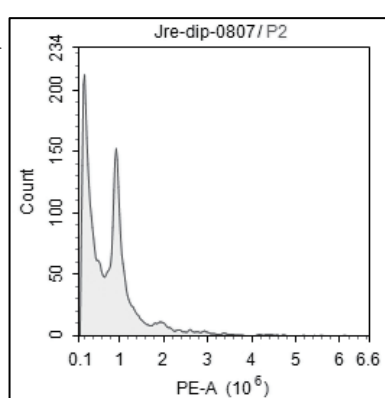**B**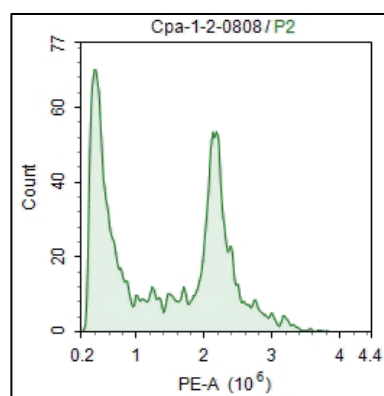**C**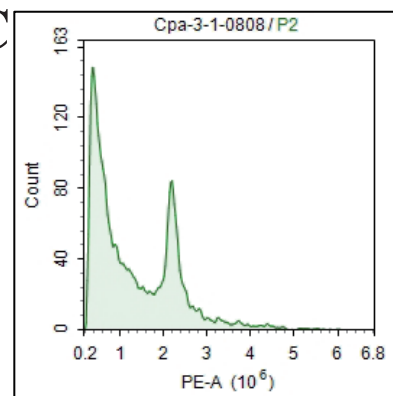**D**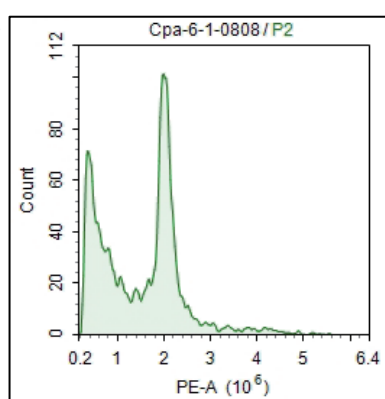**E**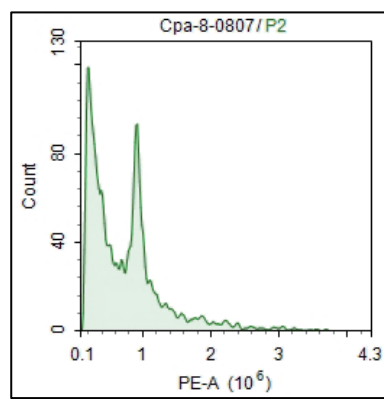**F**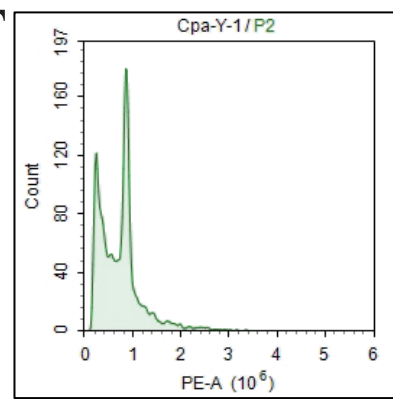**G**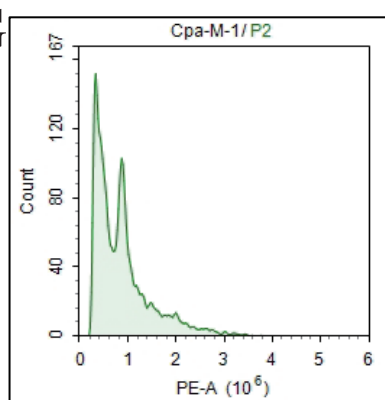**H**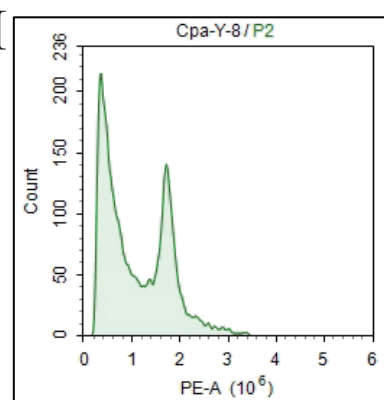**I**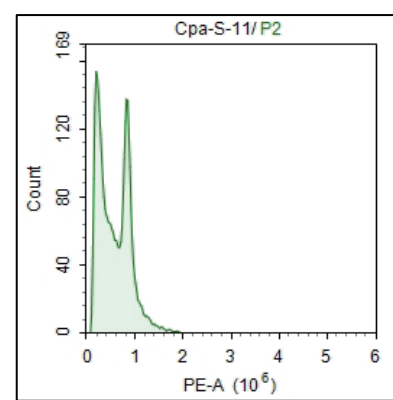**J**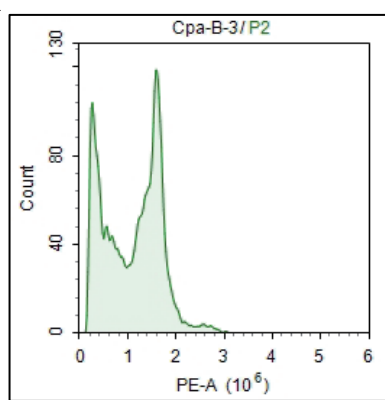**K**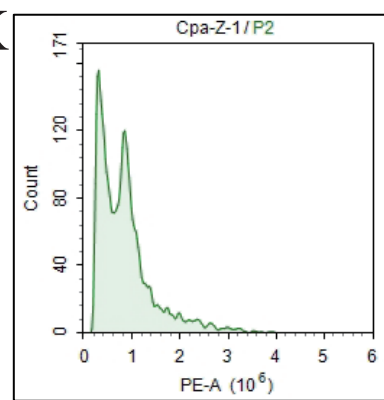**L**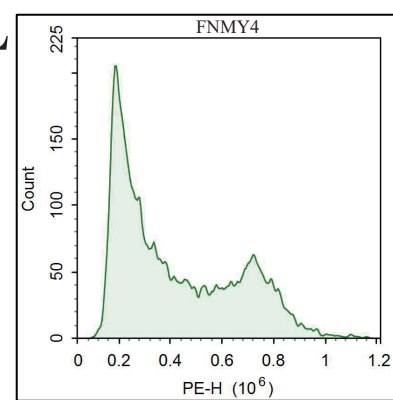**M**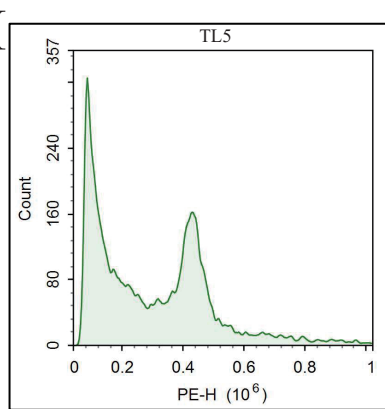

Supplement: Supplementary material 5 — Flow cytometric histograms showing the relative PI fluorescence intensity in nuclei from leaves of 12 Cyclocarya samples, with J. regia as the internal reference [file phytokeys-262-045_article-155490__-s005.pdf]

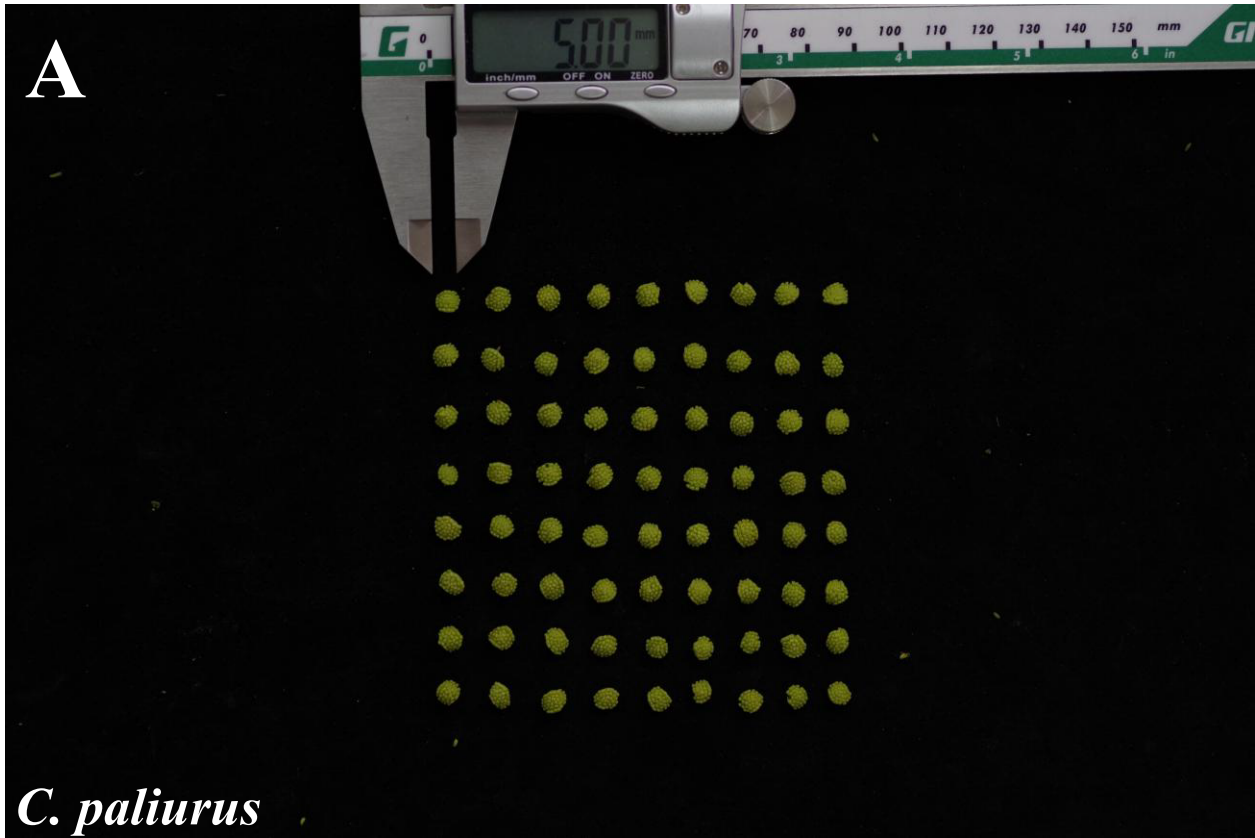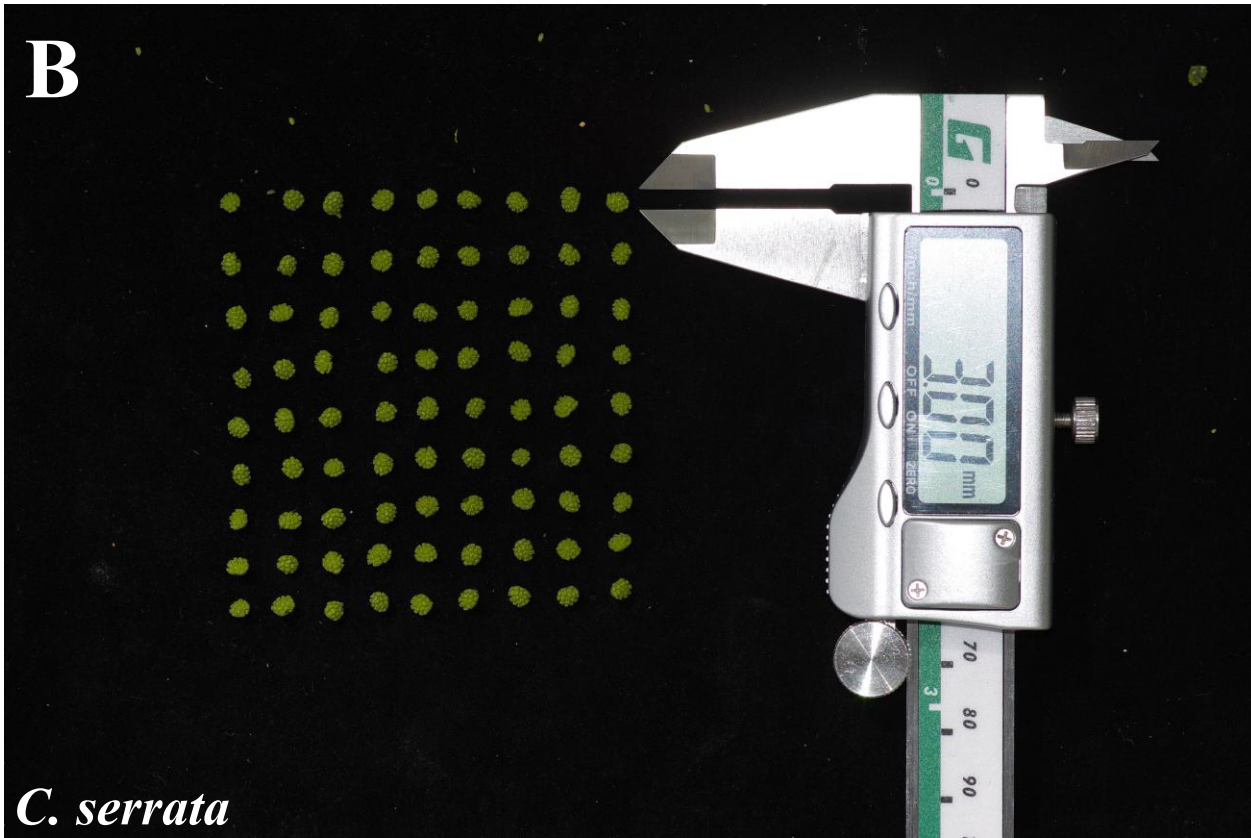

Supplement: Supplementary material 8 — Male flowers of C. paliurus and C. serrata [file phytokeys-262-045_article-155490__-s008.pdf]

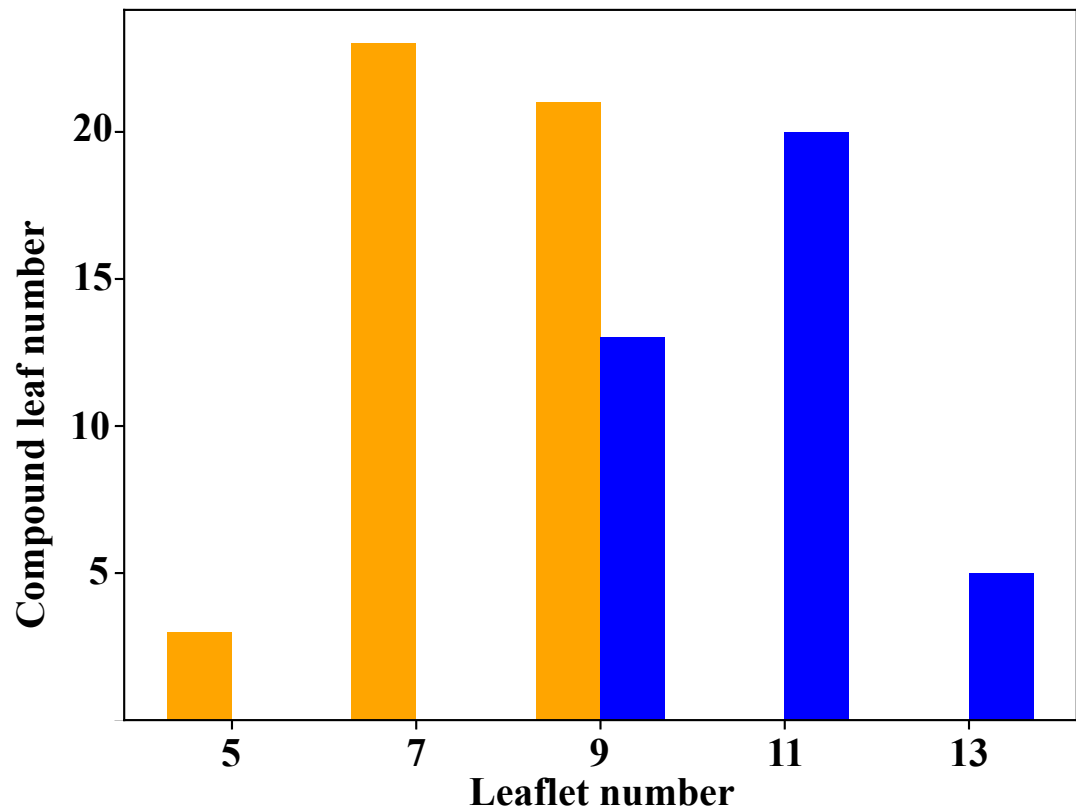

Supplement: Supplementary material 10 — Bar chart of leaflet numbers, with orange representing C. paliurus and blue representing C. serrata [file phytokeys-262-045_article-155490__-s010.pdf]

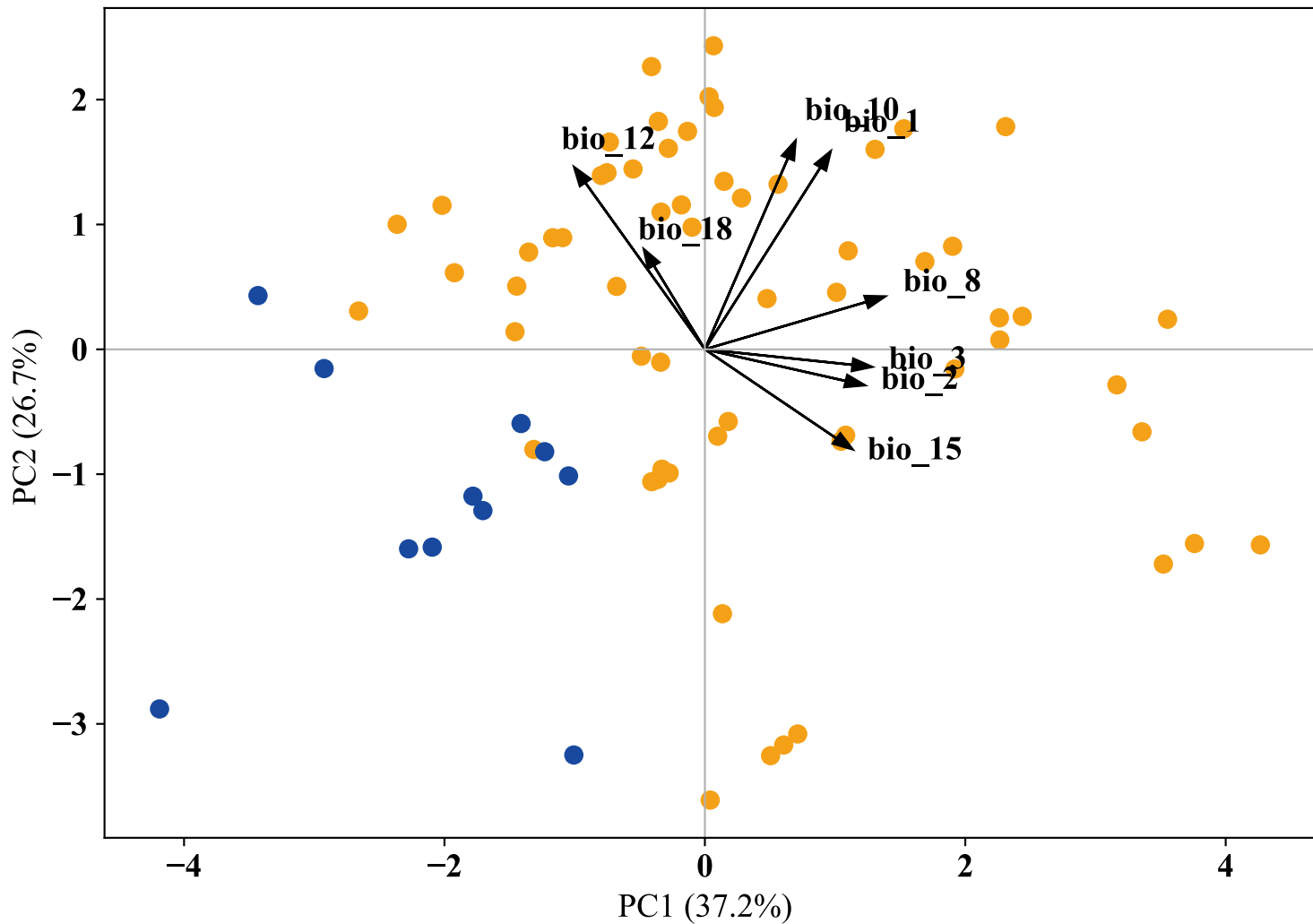

Supplement: Supplementary material 11 — PCA plot showing climatic niche differentiation between the two species [file phytokeys-262-045_article-155490__-s011.pdf]

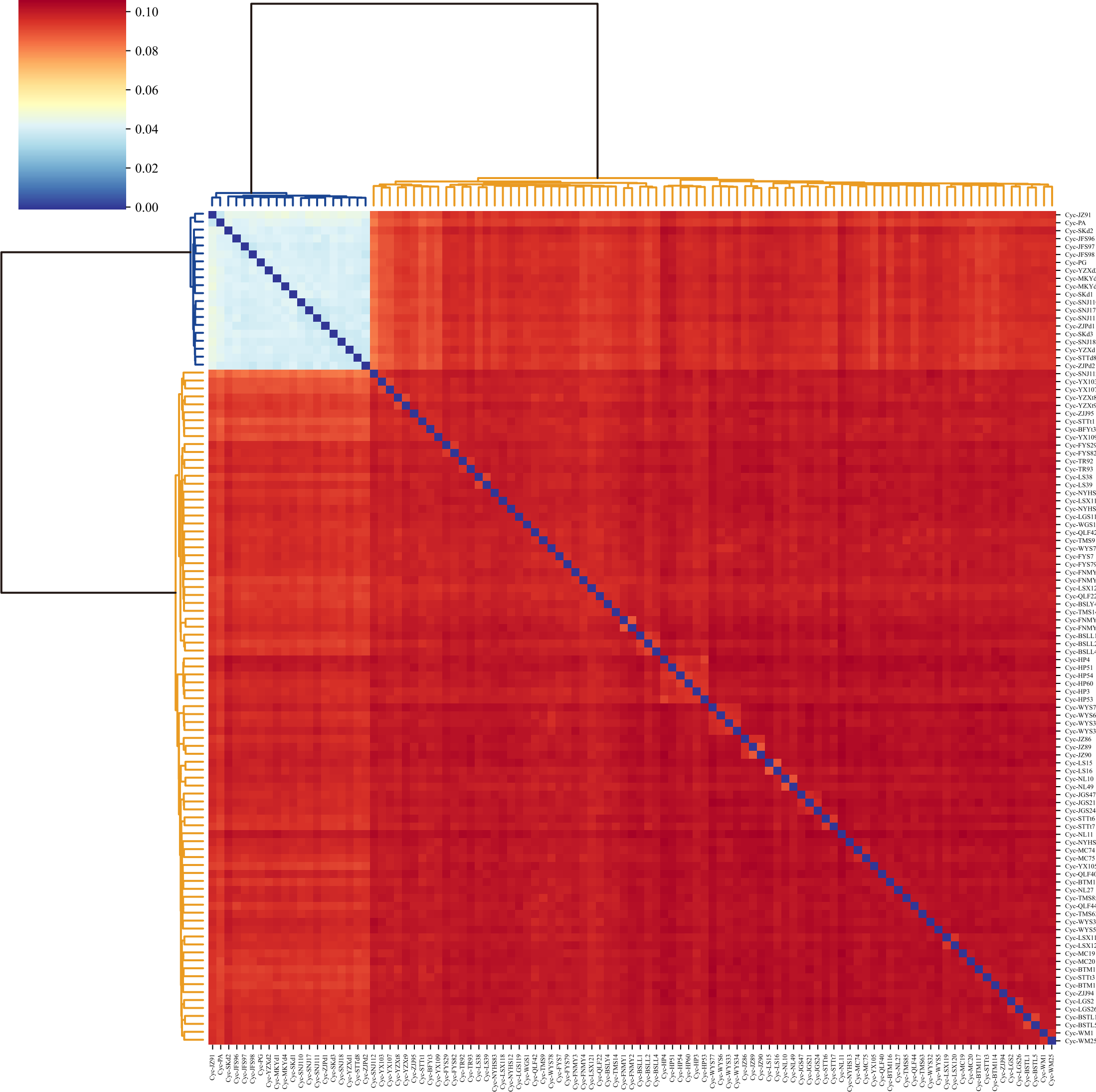

Supplement: Supplementary material 13 — Clustering heatmap based on Dxy values, with orange and blue clades representing C. paliurus and C. serrata, respectively [file phytokeys-262-045_article-155490__-s013.pdf]
